# Differentiated mental health patterns in pregnancy during COVID-19 first two waves in Sweden: a mixed methods study using digital phenotyping

**DOI:** 10.1038/s41598-022-25107-3

**Published:** 2022-12-08

**Authors:** Emma Fransson, Maria Karalexi, Mary Kimmel, Emma Bränn, Natasa Kollia, Auke Tas, Vera van Zoest, Eira Nordling, Alkistis Skalkidou, Fotios C. Papadopoulos

**Affiliations:** 1 Department of Women’s and Children’s Health, Uppsala University, 751 85 Uppsala, Sweden; 2 Department of Microbiology, Tumor and Cell Biology, Centre for Translational Microbiome Research, Karolinska Institutet, 171 77 Stockholm, Sweden; 3 Department of Hygiene and Epidemiology, School of Medicine, University of Ioannina, 45110 Ioannina, Greece; 4 Department of Psychiatry, University of North Carolina at Chapel Hill, Chapel Hill, NC 27514 USA; 5 Department of Nutrition and Dietetics, School of Health Science and Education, Harokopio University, 17676 Athens, Greece; 6 Department of Neuroscience, Psychiatry, Uppsala University, 751 85 Uppsala, Sweden; 7 Department of Information Technology, Uppsala University, 752 37 Uppsala, Sweden

**Keywords:** Public health, Psychology, Health care, Risk factors, Signs and symptoms

## Abstract

To utilize modern tools to assess depressive and anxiety symptoms, wellbeing and life conditions in pregnant women during the first two waves of the COVID-19 pandemic in Sweden. Pregnant women (n = 1577) were recruited through the mobile application Mom2B. Symptoms of depression, anxiety and wellbeing were assessed during January 2020–February 2021. Movement data was collected using the phone’s sensor. Data on Google search volumes for “Corona” and Covid-related deaths were obtained. Qualitative analysis of free text responses regarding maternity care was performed. Two peaks were seen for depressive symptoms, corresponding to the two waves. Higher prevalence of anxiety was only noted during the first wave. A moderating effect of the two waves in the association of depression, anxiety, and well-being with Covid deaths was noted; positive associations during the first wave and attenuated or became negative during the second wave. Throughout, women reported on cancelled healthcare appointments and worry about partners not being allowed in hospital. The association of mental health outcomes with relevant covariates may vary during the different phases in a pandemic, possibly due to adaptation strategies on a personal and societal/healthcare level. Digital phenotyping can help healthcare providers and governmental bodies to in real time monitor high-risk groups during crises, and to adjust the support offered.

## Introduction

The coronavirus disease 2019 (COVID-19) has led to a huge healthcare crisis with significant sequelae; the global death toll is close to four million and the number of confirmed cases more than 188 million people^1^. The effects of COVID-19 posed significant threats to mental health with recent studies reporting an increase by 15–30% in anxiety and depression globally; from experiences such as grief and isolation along with the infection itself having effects on the nervous system and resulting symptoms of mental illness of unknown duration^2–5^.

Peripartum depression and anxiety have far-reaching effects, including severe consequences on the mother’s future morbidity and the fetus’s psycho-emotional development^6,7^. Among pregnant women, recent studies in the North America, China, Japan and Turkey have shown an increase by 25–50% in peripartum anxiety and depressive symptoms since the beginning of the pandemic^2,8–14^. Worry for the health of the pregnancy and the forthcoming infant, impacts on healthcare services, and general impacts of the pandemic including economic consequences, grief for victims, misinformation about the virus, social distancing/social isolation and travel restrictions could underlie this increase^2,3,15–17^. In relation to the provision of healthcare services, the epidemic has greatly affected hospital organization and procedures related to childbirth^18^.

Sweden, with 115,000 births per year, has one of the lowest levels of maternal and neonatal morbidity and mortality worldwide^19^. Sweden is also one of the countries that did not implement strict lockdown measures, but relied on voluntary social distancing guidelines, including working from home when possible, reducing travel and social contacts and avoiding public transport^20^. Sweden had a greater death toll than surrounding countries who did institute mandatory lockdowns^21^. Related deaths peaked in late April during the first wave, and in the second wave cases surged again in October and peaked in December 2020. The Swedish approach not to implement strict lockdown measures has garnered debate and may have resulted in greater or less anxiety, particularly for pregnant women.

Smartphone use habits have been shown to correlate with mental illness. Thus, novel ways of collecting such data, so-called digital phenotyping, are expected to constitute a complementary piece of the puzzle, further enhancing the prediction methods for perinatal mental illness, especially in light of the disruption of normal daily life patterns due to the COVID-19 pandemic^22,23^. Of note, as many as 92% of the Swedish population above the age of 12 owned a smartphone in 2019.

The Swedish background of low levels of maternal and neonatal morbidity and relative economic stability, in combination with the absence of mandatory lockdown measures, provide a unique setting to study the impact of COVID-19 on perinatal depression and anxiety as measured using digital phenotyping. We utilized data from a unique cohort of pregnant women recruited for research using the novel Mom2B mobile application, with the aim to examine: (1) how measures of mental health fluctuated during the first two waves of COVID-19 in Sweden and compared with before the pandemic, (2) if mobility, COVID-19-related internet searches and COVID-19 reported deaths correlated with mental health symptoms, (3) if the waves moderated potential associations, (4) whether confirmed or possible infection and/or other self-reported impact from the pandemic were associated with mental health, and finally (5) which types of concerns were noted by Swedish pregnant women specific to their health care and wellbeing during the pandemic.

## Methods

### Study population and procedures

The Mom2B cohort (http://www.mom2b.se) is a national ongoing mobile application-based study for pregnant and postpartum individuals; the application was introduced at the end of November 2019 to the App Store and Google Play. All Swedish-speaking women above 18 years of age owning a mobile smartphone who are either pregnant or have delivered within 3 months are eligible for participation. Information about the study is being posted on social media, as well as by using posters and brochures at local maternity clinics. The Mom2B application collects three types of data from early pregnancy up until 1 year after birth: survey data, audio recordings, as well as passive data, such as mobility. The application delivers a range of both validated and self-developed questionnaires two to three times per week on average, with a mean of five questions per survey. The study protocol has been presented previously in detail^24^.

By the end of February 2021, 1577 had answered at least one questionnaire related to mental health measures during pregnancy. The included self-report instruments were based on the results from a previous study in Uppsala County, Sweden^25^, whereas the mobile application further recorded digital phenotyping data, such as movement patterns, internet and mobile use, as well as voice recordings, after receiving informed consent from participants. Privacy protection was central in the design of the application and data was collected only on relative geographical movement patterns, i.e., not the exact position of participant. The application was a further development of the Beiwe research platform from the Harvard School of Public Health, adjusted to the Mom2B study questions and adhering to the General Data Protection Regulation (GDPR) regulations in Sweden^26^.

### Comparison periods

This study investigates the two first waves of the pandemic in Sweden, using data from (1) January 2020–Sept 2020 and (2) October 2020–February 2021.

To compare the population in the Mom2B study to pre-pandemic years, we used data from 4879 pregnant women recruited in the Biology, Affect, Stress, Imagine, and Cognition (BASIC) study^25^, a population-based longitudinal cohort study, conducted in Uppsala County during 2009–2019. Participants in the BASIC study answered web-based self-report instruments regarding mental health.

The studies have similar design and participant characteristics, besides the nation-wide nature and mobile use in the Mom2B study.

### Ethical considerations

All methods were performed in accordance with the Declaration of Helsinki and ethical approval for the BASIC study was granted by the Regional Ethical Review Board of Uppsala, Sweden (Dnr 2009/171), and for the Mom2B-study by the Swedish Ethical Review Authority (Dnr 2019-01170), with amendments. Informed consent was obtained from all participants. Separate consents were obtained for mobility data, survey data etc. Only data that participants consented for were collected and participants can change their consent preferences anytime in the app if they wish to stop. The data are stored pseudoanonymised on secure servers, as previously described^24^.

### Variables of interest

Perinatal depressive symptoms and anxiety were assessed with the Edinburgh Postnatal Depression Scale (EPDS)^27^ a validated ten-item self-report questionnaire assessing depressive symptoms in the perinatal period with good psychometric properties^28^. For the present study, a cutoff point of greater than or equal to 13 points was used to define probable cases of depression during pregnancy^29^. Three items were used for assessing symptoms of anxiety (EPDS-3A)^30^ with a cut-off score greater than or equal to 7 points^30^. The EPDS was collected in the Mom2B study at the end of the first, second and third pregnancy trimester.

The second self-report inventory was the five-item World Health Organization wellbeing index (WHO-5)^31^ that investigates the degree of subjective quality of life based on positive mood (good spirits, relaxation), vitality (being active and waking up fresh and rested), and general interest (being interested in things). Each item is rated from 0 to 5; the total score ranged from 0 to 25. According to the instructions, a percentage value was calculated by multiplying the score by 4 and thus obtaining a scale from 0 (worst) to 100 (best). A percentage score below 50 is interpreted as indicating risk of depression^31^.

The following questions concerning the impact of the pandemic were assessed three times during pregnancy between gestational weeks 11–20, 22–30 and 32–42:

1. *Have you had symptoms similar to the description of Covid-19?* (a) Yes, tested positive; (b) Yes, tested negative; (c) Yes, was not tested; (d) No, but someone in my family had symptoms and (e) No, and no one in my family had any symptoms.
2. *How is your life situation affected by the pandemic?* (a) It is not affected; (b) Only slightly affected; (c) There is a lot in my life that is affected and (d) Almost everything in my life is affected.
3. *Which option describes your current situation?* (a) My situation is about normal; (b) I am more isolated, and I am negatively affected; (c) I am more isolated, but I feel OK and (d) I am more isolated and this is mostly positive for me.

Confirmed or possible Covid-19 infection was defined as the participant replying with one of the following: 1a, 1b, 1c. Life situation was defined as “affected” when the participant replied with 2c, 2d or 3b. There was also an open question regarding the potential impact of the pandemic on maternity and delivery health care received.

### COVID-19 related measures

Data on COVID-19 confirmed cases and COVID-19-related deaths were downloaded from the Statistics Sweden website (https://www.scb.se/en/finding-statistics/coronavirus/). In our analyses, only data on COVID-19-related deaths were used as proxy of the pandemic’s impact during the two waves, as testing capacity and thereafter registered confirmed cases varied substantially between the first and the second wave.

### Mobility data

Movement data was collected using the GPS sensor on the phone. The GPS sensor was iteratively turned on for 60 s and turned off for 10 min. A random offset between − 100 and + 100 km was applied in both latitude and longitude to preserve the privacy of the participants. In total, GPS data was available for 1885 participants. For each participant, records before January 2020 and after February 28, 2021 and records with a high inaccuracy (> 100 m) were removed. Participants were also removed if they had less than 100 records remaining. After data cleaning, 1575 participants were included in this analysis.

Since the GPS sensor is turned on and off with a fixed interval, the absolute distances travelled for each user every month could not be calculated; instead, the relative difference in distance travelled away from their relative “home position” between months was used. The approximate home location of each participant was estimated by taking the median of the latitude and median of the longitude, assuming that each participant spends the majority of her time at home, and adding a radius of 100 m. Next, for each GPS record outside the “home location”, the Euclidean distance to the home location was calculated. For each participant, the monthly median of the distance travelled away from home was used. The aggregated between-participant summary statistics were computed over these monthly medians.

### Google search volumes data

The interest of internet users for the situation around the pandemic over time (January 2020-February 2021) in Sweden was assessed using Google Trends (https://trends.google.com/trends/explore?geo=SE&q=Corona). The numbers procured represent search volumes relative to the highest point on the chart (week with highest number of searches) for the given region and time. A value of 100 denotes the timepoint for the peak popularity for the term. A value of 50 means that the search term is half as popular as during the peak interest period. A score of 0 means there was not enough data for this term.

### Statistical analyses of quantitative data

Descriptive statistics for continuous variables were presented using median and range (min–max) while for the categorical variables the absolute and relative frequencies (n, %) were calculated. Prevalence of Depression (EPDS total score ≥ 13), Anxiety (EPDS-3A total score ≥ 7) and Low Well-being (WHO-5 total score < 50) for each study period and the monthly prevalence of depressive symptoms, based on the EPDS, during the previous years (BASIC study data 2010–2019) were calculated. Differences in the above measurements by time period were assessed using Pearson’s chi-square statistic (Aim 1). Additionally, depressive symptoms before the Corona pandemic (BASIC data), during Jan-Sep 2020 (Mom2B data) and Oct 2020–Feb 2021 (Mom2B data) and differences in anxiety symptomatology and low well-being prevalence between wave 1 and wave 2 were compared by the use of Pearson’s chi-square test (Aim 1). Pearson’s correlation coefficients and the respective *p*-values were calculated, separately for each wave, to assess possible associations between the monthly and weekly mean total scores in EPDS, EPDS-3A, WHO-5 and (a) the monthly median distance away from home, as well as with (b) weekly relative google search volumes on “corona” and (c) number of weekly deaths attributed to COVID-19 (Aim 2). The corresponding scatterplots stratified by wave were also created, as well as a graphical presentation of each variable over time. The associations between more clinical levels of depression, anxiety and low-wellbeing and mobility, google search volumes and COVID-19 deaths were examined with linear as well as logistic regression models, using known cutoffs in the logistic regression models for depression (EPDS ≥ 13), anxiety (EPDS-3A ≥ 7) and low wellbeing (WHO-5 < 50) to calculate the percentage above the cutoffs as the outcome variable for the model (Aim 2).

The moderating effect of the different pandemic waves on the associations between the outcome measures and Google internet searches and COVID-19 deaths was examined as interaction terms in linear regression models, where the outcome measures were the scores in depressive, anxiety and low wellbeing symptoms and the explanatory variables were google searches or COVID-19 related deaths, a binary wave-specific variable and the interaction term between Google searches or COVID-19 relates deaths and the wave. Similarly, the moderating effect of different pandemic waves in the more severe clinical (above a certain threshold) of depression, anxiety and low wellbeing was examined with interaction terms in the logistic regression models described above (Aim 3).

Further exploration on a possible lag effect of the reported COVID-19 deaths and google searches was done by performing the above analyses using reported COVID-19 deaths and Google searches during the previous week.

Responses on questions concerning the COVID-19 pandemic at early (11–20 gestation weeks), middle (21–30 gestation weeks) and late (31–42 gestation weeks) pregnancy for each wave were presented and compared between waves using the Pearson’s chi-square test (Supplementary Table 1). Associations between the prevalence of Depression, Anxiety and Low well-being (at early-middle-late pregnancy) and the self-reported impact of the pandemic were also assessed using the Pearson’s chi-square test (Supplementary Table 2) (Aim 4). All reported *p*-values were based on two-sided tests and the 5% significance level was used for hypothesis testing. All data were pseudoanonymised and analyzed on group level. Analyses were performed using the SPSS software, version 26.0 as well as R.

### Qualitative data and analysis

Analysis of the free text responses was performed using systematic text condensation^32^ to comprehend the health care experiences of pregnant women during Covid-19 pandemic for the two waves (Aim 5). Every answer was divided into “meaning units” and the main content of the meaning units were identified and categorized. Notes were made regarding what time period the identified categories were represented by comments from the participating women (or if represented at both time points).

## Results

### Background characteristics

The background characteristics of the 1577 women included in the Mom2B-study are presented in Table 1. The majority of participants were born in Sweden (93%), were primiparous (53%), had a university level education (74%) and lived with a partner (97%). More than half of women reported having experienced depression earlier in life (Table 1).

**Table 1:** Background characteristics of the participating women in the Mom2B-study (n = 1577).

Characteristics		Valid, n
Mother's age in years, median (range)	32 (19–46)	1454
BMI^a^ in kg/m^2^, median (range)	24.1 (16.7–63.7)	1424
Primipara, n (%)	770 (53%)	1463
Born in Sweden, n (%)	1353 (93%)	1460
**Educational level**	n (%)	1461
Primary school	29 (2%)	
High school	234 (16%)	
Polytechnic or vocational training	122 (8%)	
University or college	1076 (74%)	
**Relationship status**	n (%)	1463
No	26 (2%)	
Yes, I have a partner and we live together	1416 (97%)	
Yes, I have a partner but we do not live together	21 (1%)	
**Depression history before pregnancy**	n (%)	1443
No	657 (46%)	
Yes, and I got help from a psychologist/psychiatrist/counselor	654 (45%)	
Yes, but I did not seek or receive any professional help	132 (9%)	
Smoking during the last 3 months before pregnancy, n (%)	195 (14%)	1423
Mobility^b^ in kilometers, median (range)	5.0 (3.4–21.1)	1557

^a^Body Mass Index.

^b^Distance travelled away from home per month.

### Mood symptoms over the first two waves of the pandemic (Aim 1)

**Figure 1:**
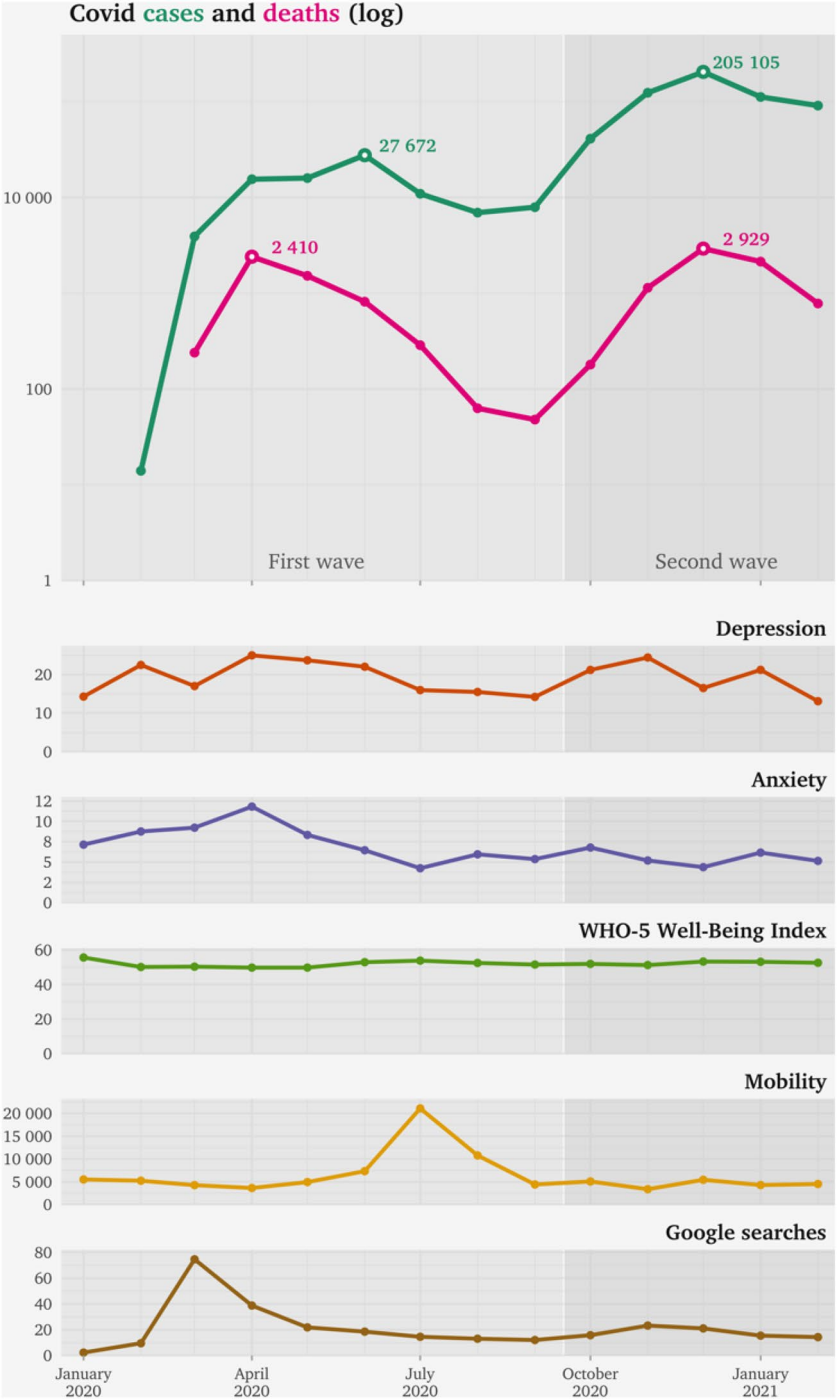
The figure presents log transformed number of cases with confirmed COVID-19 infection and deaths in Sweden from January 2020-February 2021 (top panel). The lower panel shows the proportion of women over the clinical cut-offs regarding symptoms of depression on the Edinburgh Postnatal Depression Scale (EPDS) and anxiety measured by three questions on the same scale (EPDS-3A), followed by the proportion of women with high wellbeing according to the Well-being index measured with the WHO-5, mobility index (the monthly median of the median distance travelled away from home /100) and google searches performed in Sweden for “Corona”, where 100 denotes the timepoint for the peak popularity for the search term and 50 means that the term is half as popular.

As illustrated in Fig. 1, the proportion of women with depressive symptoms over the clinical cut-off showed a clear peak in each of the two waves, (25% in April 2020 and 24% in November 2020), with an average (shown in Table 2) of 18.3% during the first wave and 20.1% during the second wave. Those numbers were significantly increased compared to the average of 8.0% in the period of 2010–2019 (p < 0.001, see Table 2) when comparing with data from previous years^20^. A significantly lower prevalence of anxiety (p = 0.023) was noted during the second wave compared to the first wave of the pandemic (Fig. 1 and Table 2).

**Table 2:** Prevalence of positive screening for depression, anxiety and low wellbeing using the Edinburgh Postnatal Depression Scale (EPDS) and the WHO-5 index for the two periods during the pandemic (February-September 2020 and October 2020-February 2021) and comparisons with positive screening for depression during the period 2010–2019 from a previous study cohort.

	Before the COVID-19 pandemic (2010–2019)	WAVE 1 Jan–Sep 2020	WAVE 2 Oct 2020–Feb 2021	*p*-value^a^
Depression^d^ (%)	8.0%	18.3%	20.1%	** < 0.001**^b^**; 0.346**^c^
Anxiety^e^ (%)		6.9%	4.4%	**0.023**
Low wellbeing^f^ (%)		48.8%	51.4%	0.266

Statistically Significant values are given in bold.

^a^Chi-square derived *p*-value representing comparison of average proportions.

^b^Comparisons between each wave and the average 2010–2019.

^c^Comparisons between each wave or each wave and the average 2010–2019.

^d^Based on an EPDS total score ≥ 13.

^e^Based on an EPDS-3A total score ≥ 7.

^f^Based on a WHO-5 total score < 50.

### Google searches, number of deaths due to the pandemic and individual mobility in association with mental health symptoms

The google searches regarding the corona pandemic peaked in March, closely before the peak in symptoms of depression and anxiety and showed a slight increase starting in late October 2020 (Fig. 1). Google searches were positively associated with low wellbeing only during the first wave (R = 0.36, p-value = 0.03), no associations were found with depressive or anxiety symptoms.

**Figure 2:**
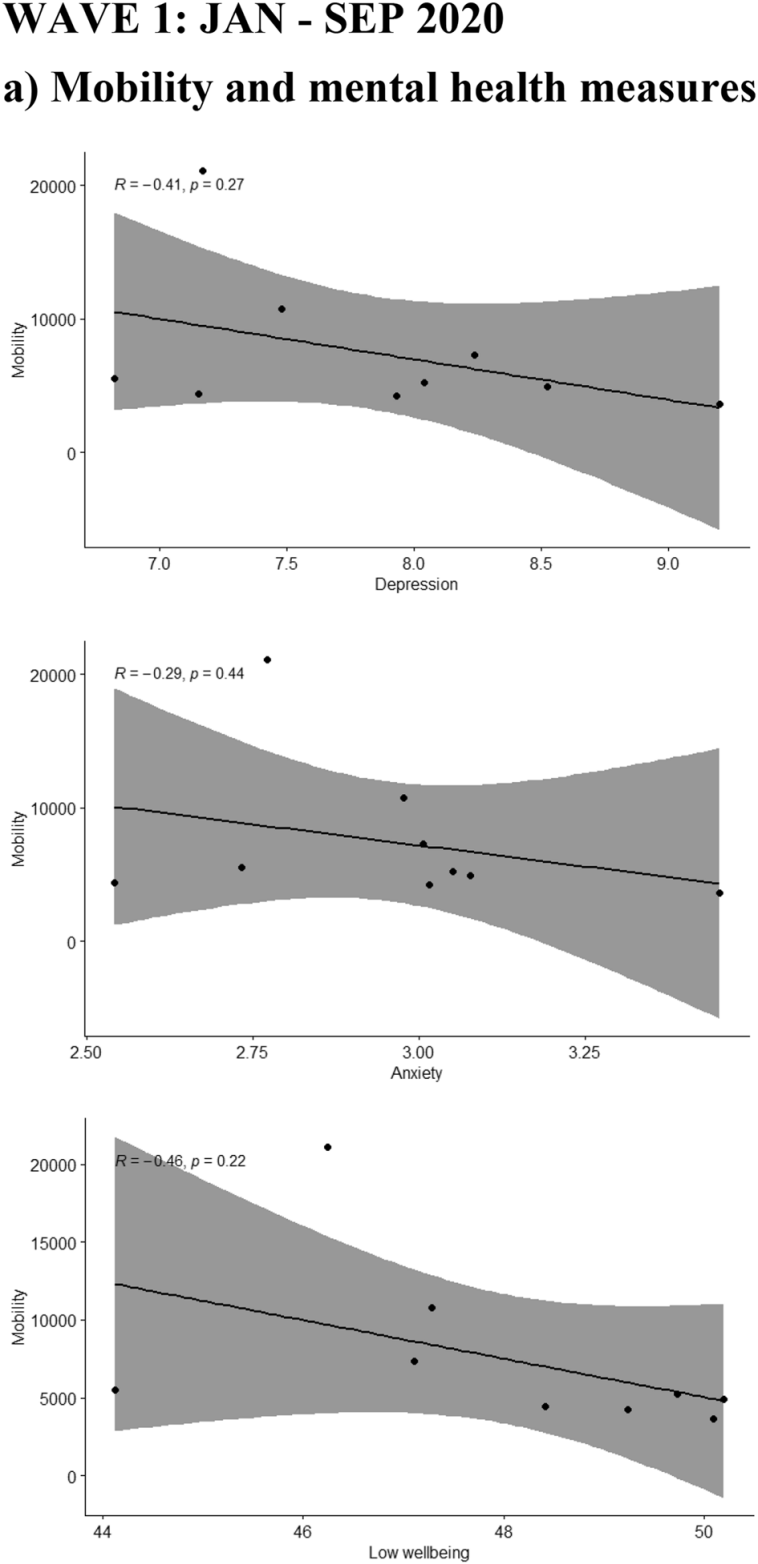

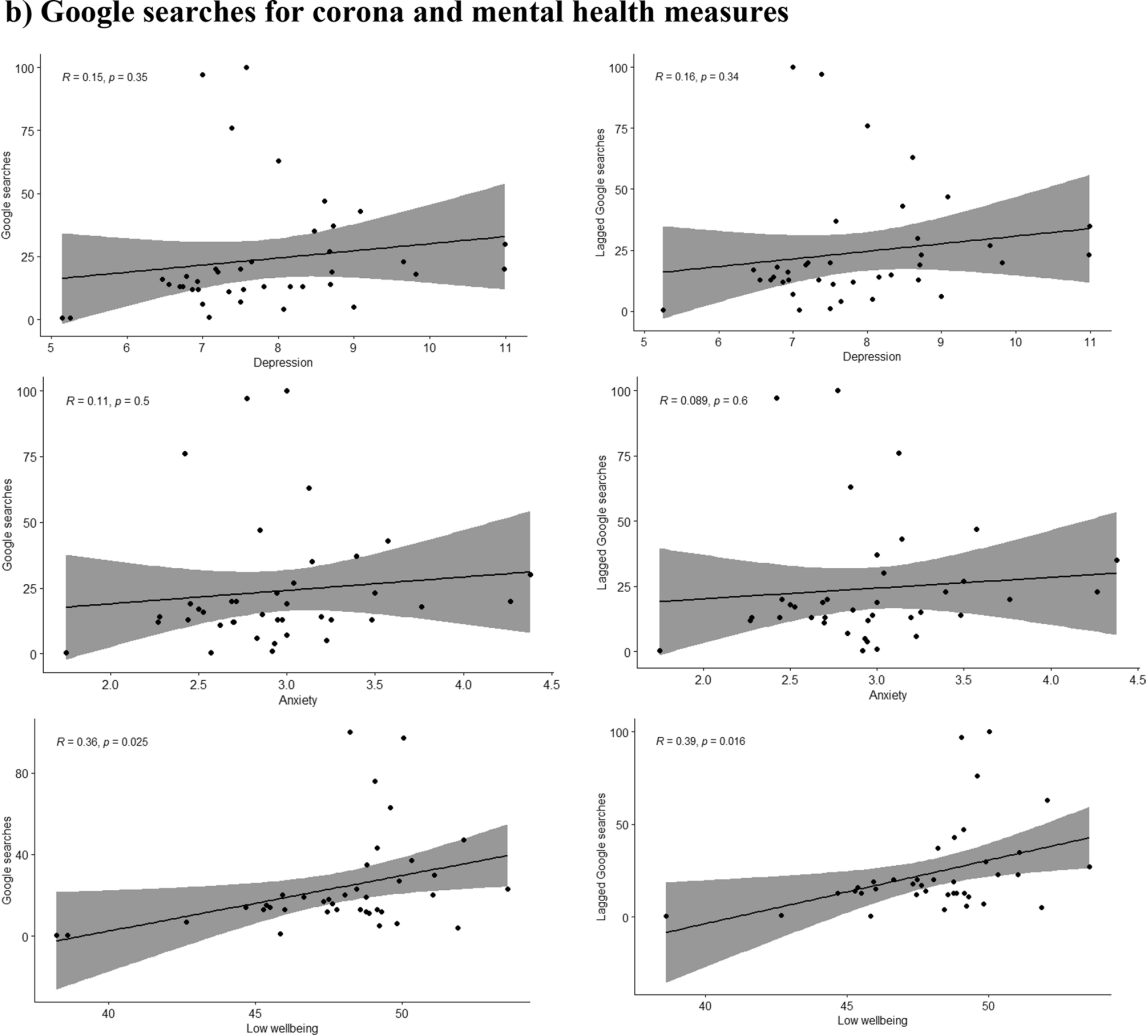

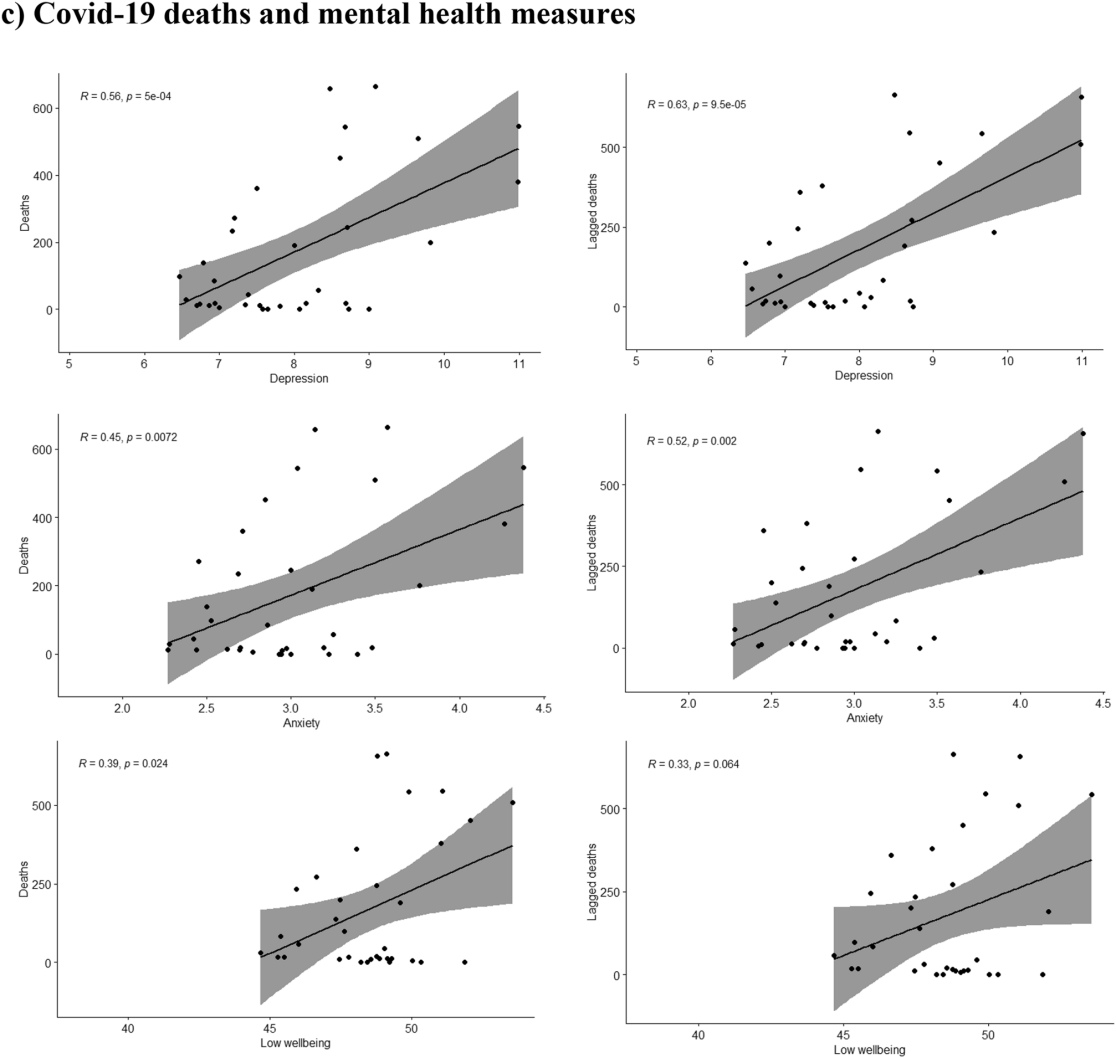

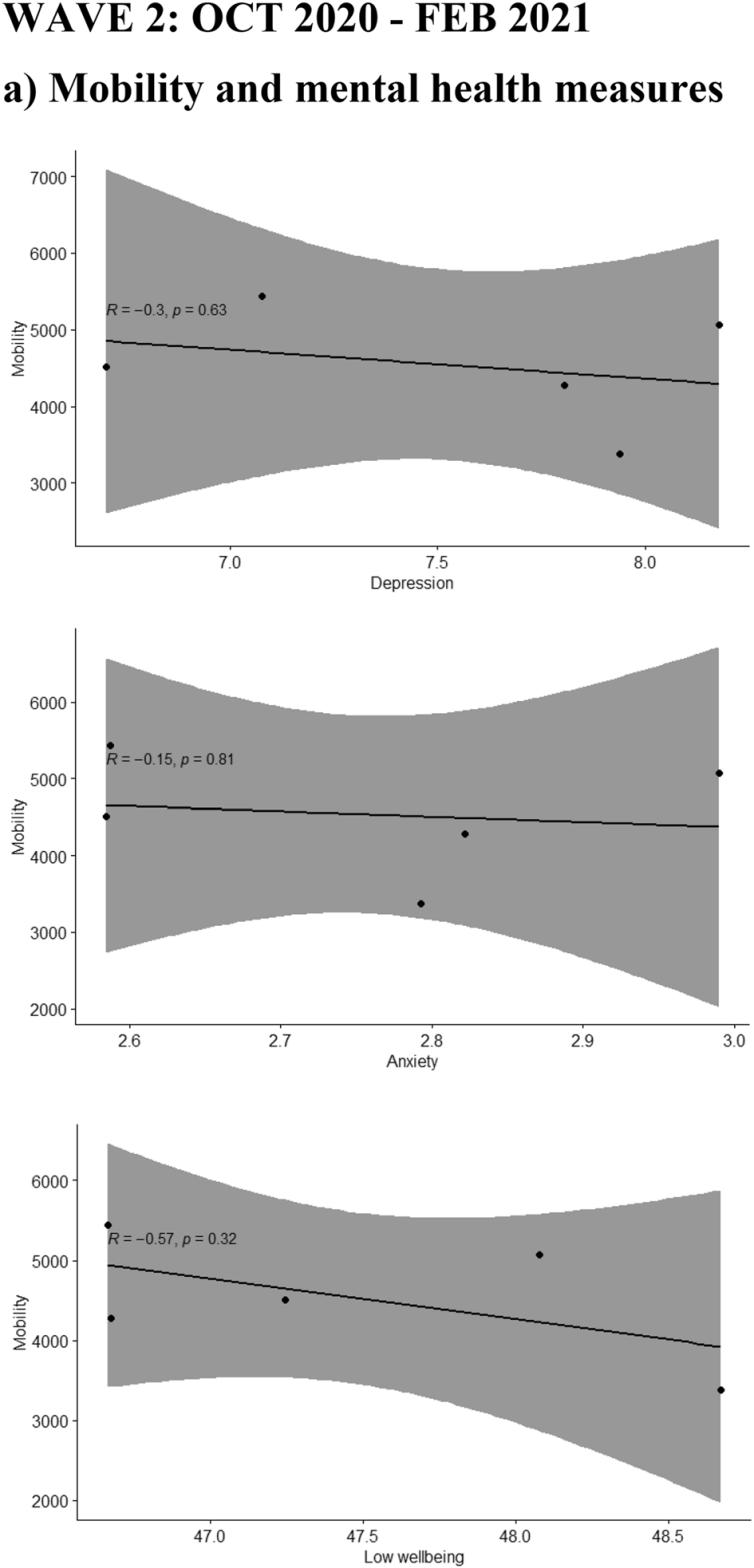

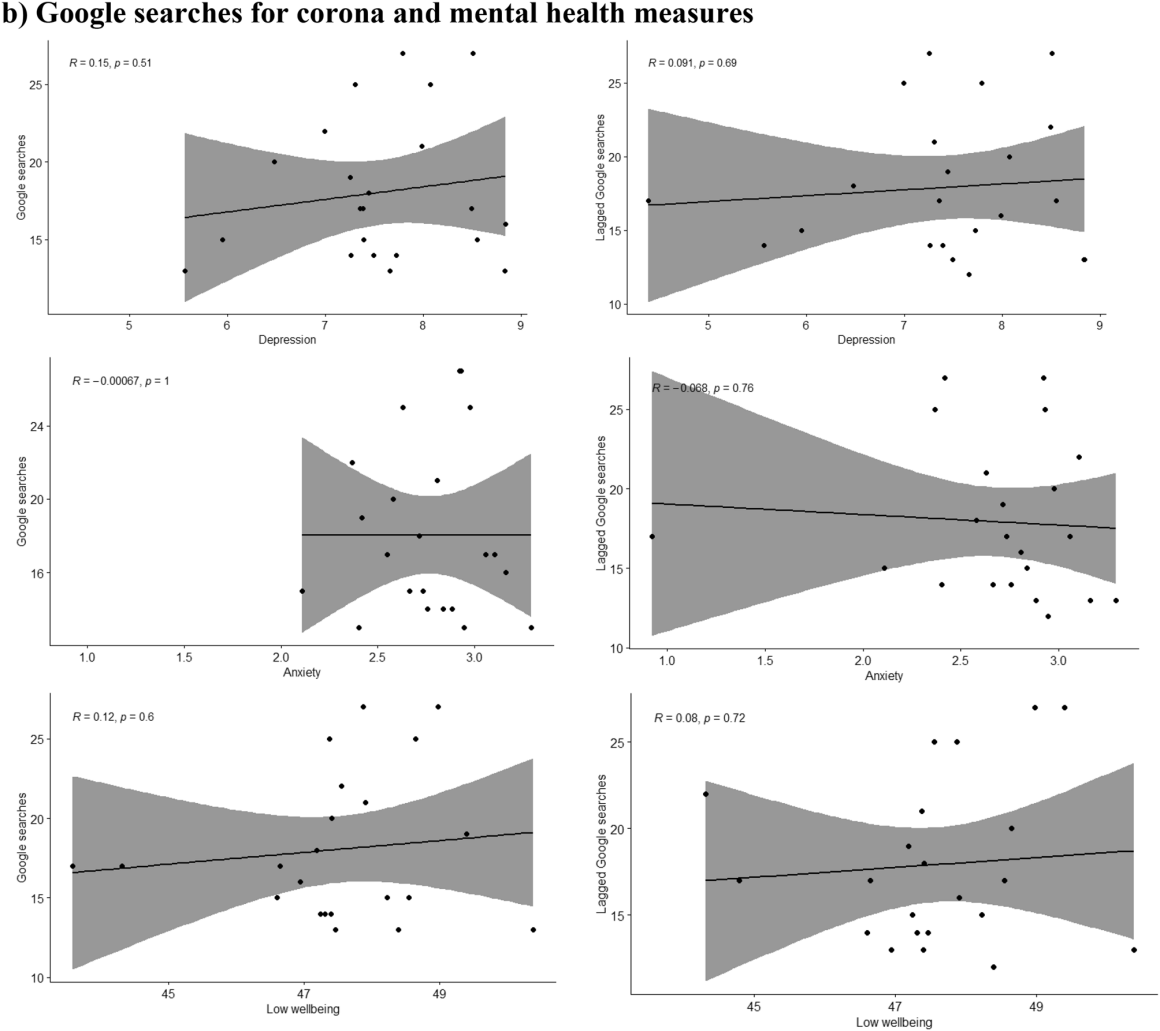

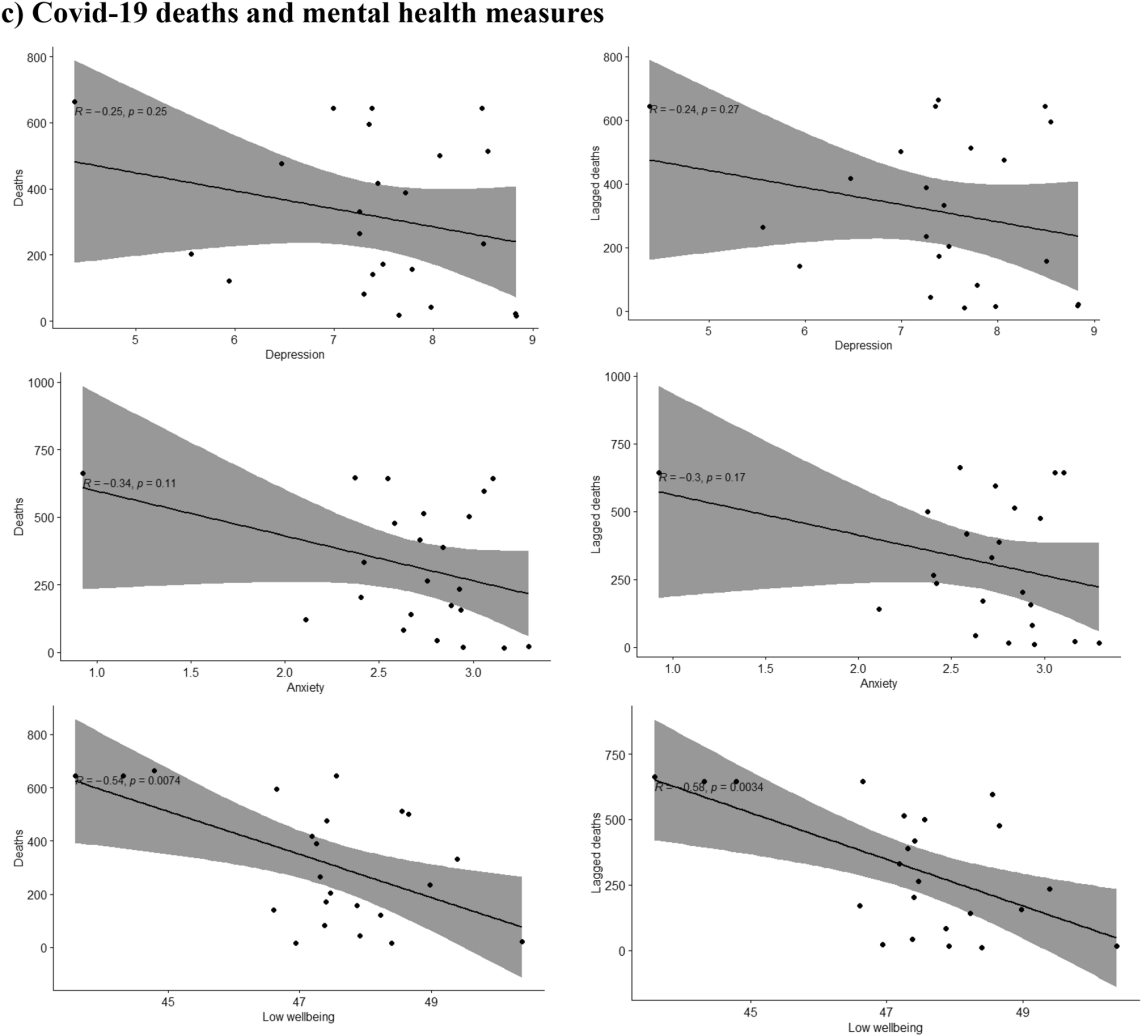
Scatterplots where the mean of Edinburgh Postnatal Depression Scale (EPDS) (depression), EPDS-3A (anxiety) and WHO-5 (wellbeing) are plotted with (**a**) mobility (monthly median distance away from home), (**b**) Google searches (weekly relative interest in the pandemic based on searches of “corona”) during the same and the previous week (lagged searched), and (**c**) weekly number of deaths (attributed to COVID-19) during the same and the previous week, separately in wave 1 and wave 2 of the pandemic.

A moderating effect of the different waves was seen for the association of depression, anxiety and low wellbeing with the reported COVID-19 deaths during the same (p-values for the interaction terms 0.001, 0.003 and 0.001 respectively) or the previous week (p-values < 0.001, 0.002 and 0.001 respectively). The moderate positive associations between depression, anxiety and low wellbeing with COVID-19 deaths were only seen during the first wave of the pandemic but not during the second wave, as shown in Fig. 2. During the second wave the associations were either attenuated or inversed and in the case of low wellbeing, a moderate negative association with lower wellbeing associated with lower COVID-19 deaths during the same or the previous week as shown in Fig. 2.

Mobility peaked during the summer month of July 2020 (Fig. 1) and did not show significant correlations with the mental health outcomes.

No associations were found when the outcomes of depression, anxiety or low wellbeing were examined as prevalence of symptoms above our chosen cutoffs, neither in total or in specific waves.

### Life situation during the pandemic

There were differences in the reports regarding life situation of the participating pregnant women between the two waves. During the first wave, more women reported on close relatives having symptoms that resembled COVID-19 (Supplementary Table 1). However, during the first wave, 24–32% (depending on pregnancy trimester) reported no social isolation, compared to 13–19% during the second wave. More women in the second wave reported being socially isolated in a negative way (42–48%, compared with 29–35% during the first wave). The proportion of women who experienced that a lot or almost everything was affected by the pandemic, increased from 46–53% to 54–59%, however not significantly, see Supplementary Table 1.

A higher proportion of women reporting symptoms indicating COVID-19 reported lower wellbeing, a pattern consistent in both waves (Supplementary Table 2), but no consistent pattern was observed between COVID-19 symptoms and the prevalence of depressive or anxiety symptoms. Similarly, women reported that their life situation was greatly impacted by the pandemic had lower wellbeing in both waves, with no consistent pattern of increase of depressive or anxiety symptoms. On the contrary, a higher prevalence of depressive symptoms was observed among those women (approximately 40%) who reported social isolation which they found difficult.

### Participants experiences of the perceived heath care during the pandemic

Table 3 presents a compilation of the women’s free responses to the open questions regarding healthcare during the pandemic across pregnancy and childbirth. The answers have been divided into different categories based on first and second wave time periods or both, and representative quotes noted. The participants expressed loss of support when partners were excluded from attending visits at the maternity center and ultrasound examinations. Many comments from the first and second wave addressed the risks of the partner missing out during the delivery of the child, as no-one with even slight symptoms could accompany the delivering woman. Moreover, other support stemming from relatives or doulas was not allowed, which further enhanced worry and distress. When participants mentioned that check-ups had been canceled/postponed, they usually referred to ultrasound examinations or combined ultrasound and biochemical screening. Moreover, they also reported meetings with other professionals, such as social workers postponed or cancelled. Some participants also felt unsafe about their health care. They experienced miscommunication and insufficient information from health care professionals. Referrals had been rejected due to the strain caused by COVID-19. Some comments referred to the notion that online or phone meetings were not satisfactory regarding the level of care. During the second wave, the negative impact on communication by staff due to wearing face masks and visors was noted.

**Table 3:** Categorization of participants' experiences of how maternity care was affected during the two first waves of the pandemic.

Categories	Quotations for illustration	Occurred during pandemic wave (1 and/o 2)
**The quality of care**	“Due to restrictions about physical appointments, I am not sure that I got the correct diagnose.” “Appointments over the phone do not provide as good care ” “My trust in health care is very low—also regarding their ability to make me feel safe.” “I worry about that I will be sent home earlier than normal after the delivery.”	1, 2
Lack of information		
Shortage of staff		
Inferior treatment		
Fewer physical examinations		
Postponed care		
**Childbirth**	“I now plan to give birth at home with a midwife" “There is a risk that I will be giving birth alone if my husband have symptoms. In that case I would chose to give birth at home “ "My partner may not be able to attend the childbirth at all if the slightest cold symptoms occur, so I must have back-up I will probably have to stay [without my partner] in the maternity ward, something I feel very anxious about”	1, 2
Reduced support due to visitor restriction policies		
Fewer possibilities to choose type of care		
Feelings of uncertainty		
**Maternity care**	"The early ultrasound was cancelled" “Very sad and worried, cried a lot when I found out that my partner was not allowed to come to [the test]. Made me feel really bad” "Very frustrated and worried about the restriction policy, which I experience as arbitrary and incomprehensible"	1
Reduced support for fear of childbirth/ due to visit restrictions		
Cancelled/postponed visits		
Preparatory courses cancelled		
New local routines		
Concern for Covid-19 infection		
Seeks health care to a lesser extent	"I do not seek health care unnecessarily/ for non-acute problems" “I was advised not to seek care”	1
**Social relationships**	"I worry about not being able to meet other mothers as I did with my first child"	1
Parent meeting and new contacts		
Communication	“More difficult to communicate with health care staff due to protective equipment” “The staff had to wear both facemasks and vizors, which made it very difficult to hear what they were saying”	2
No impact on health care	"All my appointments have been completed”	1

## Discussion

We used a novel study method, the research mobile application Mom2B, to assess, for the first time, in nearly real time, the potential changes in mood and behaviors during the pandemic (January 2020–February 2021) among pregnant Swedish women at national level. Depressive symptoms indicative of a depressive disorder experienced by pregnant women were more than doubled compared to data from 2010 to 2019. While both waves showed significantly increased depressive symptoms, there were differences between the two waves that were evident by digital phenotyping. An increase in anxiety symptoms was shown during the first wave of the COVID-19 pandemic in Sweden compared to the second wave. During the first wave, COVID-19 related deaths were closely related to increased symptoms of depression, anxiety and low wellbeing; those associations were either attenuated or inversed during the second wave. In addition, during the first wave, Google searches with regards to COVID-19 were associated with lower well-being. These different patterns of associations with the covariates, suggest an adaptation process over the pandemic’s different waves, a phenomenon also noted in recent studies^16,33–36^. Social isolation—perceived as difficult and reported by a substantial proportion of pregnant women (around 40%) was associated with higher prevalence of depressive symptoms during both waves; even in the absence of a strict lockdown and evidence that amount of movement from home did not impact mental health outcomes. Throughout the whole study period, the participating women reported worries related to reduced quality and availability of maternity care.

The present findings are in general congruent with recent research providing mounting evidence for an increase in the levels of anxiety and depression among pregnant women during the COVID-19 pandemic. Several studies in China, Japan, Turkey, Qatar, the US and Canada have reported that 15% to 35% of women scored high EPDS scores (above 12–13) during pregnancy and early postpartum period during the pandemic^2,8–13,37,38^. A Canadian study showed higher levels of not only depressive and anxiety symptoms, but also dissociative symptoms, symptoms of post-traumatic stress disorder, negative affectivity and less positive affectivity compared to a pre-COVID-19 cohort of pregnant women^15^. Other studies (i.e. from North America and Spain) showed higher proportion of clinical depression and anxiety symptoms during COVID-19 compared to previous time points, and increased depression was associated with factors like income disruptions, job insecurity, and difficulty with childcare^16,39,40^. Sweden did not have the same challenges with childcare and income disruptions, as the Swedish COVID-19 strategy did not include a general lockdown^20^; yet, there was a clear impact of the COVID-19 pandemic on the mental health of pregnant women. It could be important for policymakers to implement support strategies for vulnerable groups, including pregnant women in the context of the pandemic, to prevent the onset of anxiety, depression, and post-traumatic stress disorders^41,42^.

A strength of this study is the use of novel digital-phenotyping of individuals across Sweden through a user-friendly way that provided identification of changes in depression, anxiety and well-being and a better understanding of reasons behind these changes. Health-seeking behaviors, such as online searches, have been extensively analyzed for monitoring other diseases, such as seasonal influenza^43^; however, to our knowledge, such correlation, as the one found herein between Google search volumes for an infectious disease and low wellbeing, during the first wave, has never been reported before. Only during the first wave were Google searches increased and associated with lower wellbeing, indicating a role for dissemination of clear, supportive information as an important intervention in the beginning of similar events. This is further supported by the higher prevalence of anxiety during the first wave of the pandemic. In addition, the first wave was associated with qualitative statements around worry about ability to connect with other parents. The second wave was more characterized by depressive symptoms resulting from continued less movement, social interactions and communication being impacted by masks and vizors, which could imply deepened feelings of social distancing.

Among the limitations of the present study, we should first acknowledge possible selection bias: only 1% of the background eligible population contributed data; the application cannot be used by women not able to communicate in Swedish, or those not owning a mobile smartphone (8%). Nonetheless, the present results are in line with another recent Swedish study^44^. We cannot exclude a potential bias in the accuracy of the categorization of significant depressive symptoms resulting from the self-reported nature of the information gathered. The outcome assessments were based on screening questionnaires and not diagnostic tools, although we also used a high cut-off, more indicative of a clinical depression diagnosis. Data on mobility at the individual level would have allowed for more advanced analyses.

## Conclusions

Utilizing a novel application, an increase in depressive symptoms and decrease in wellbeing was observed during the months of highest pandemic impact in Sweden. Different patterns of associations with internet searches and COVID-19 deaths were observed during the different waves of the pandemic, possibly due to adaptation strategies on a personal and societal/healthcare level. Thus, findings during the beginning of a pandemic may not be valid later. As the goal of maternity care is to identify and reduce the risks of ill health, modern tools such as mobile applications are expected to be useful as other disasters occur and unfold, in order to timely implement preventive efforts.

## Supplementary Information

Supplementary Tables.

## Data Availability

Data are available on request to the corresponding author, after the requested has been approved by Mom2B study’s management board and Uppsala University’s data safety and security department.
