# Supplementary material for: Differentiated mental health patterns in pregnancy during COVID-19 first two waves in Sweden: a mixed methods study using digital phenotyping
Source: Sci Rep. 2022 Dec 8;12:21253. doi: 10.1038/s41598-022-25107-3 (PMC9731976; doi:10.1038/s41598-022-25107-3)
Supplement: Supplementary file 1 — Supplementary Tables. [file 41598_2022_25107_MOESM1_ESM.docx]

**Supplementary Table 1.** Responses on questions concerning the COVID-19 pandemic.

|  | **Early pregnancy (gw^a^ 11-20)**  n (%) | | | **Mid pregnancy (gw^a^ 22-30)**  n (%) | | | **Late pregnancy (gw^a^ 32-42)**  n (%) | | |  |  |
| --- | --- | --- | --- | --- | --- | --- | --- | --- | --- | --- | --- |
|  | Jan 2020-Sep 2020  n=244 | Oct 2020-Feb 2021  n=352 | p^b^ | Jan 2020-Sep 2020  n=418 | Oct 2020-Feb 2021  n=416 | p^b^ | Jan 2020-Sep 2020  n=320 | Oct 2020-Feb 2021  n=402 | p^b^ | |  |
| **Have you had symptoms similar to the description of Covid-19?** |  |  | **<0.001** |  |  | **<0.001** |  |  | **<0.001** | |  |
| *Yes, but I have tested negative* | 0 (0%) | 100 (29%) |  | 0 (0%) | 125 (31%) |  | 0 (0%) | 130 (34%) |  | |  |
| *No, not me and no one in my household or other close friend/relative* | 134 (55%) | 120 (35%) |  | 242 (58%) | 143 (35%) |  | 183 (58%) | 116 (30%) |  | |  |
| *No, not me but someone or more in my household or another close friend/relative* | 49 (20%) | 60 (17%) |  | 93 (23%) | 51 (13%) |  | 66 (21%) | 66 (17%) |  | |  |
| *Yes, but I have not been tested* | 55 (23%) | 45 (13%) |  | 74 (18%) | 57 (14%) |  | 66 (21%) | 48 (12%) |  | |  |
| *Yes, and I have tested positive* | 4 (2%) | 19 (6%) |  | 5 (1%) | 27 (7%) |  | 0 (0%) | 28 (7%) |  | |  |
| **Many are more socially isolated during the pandemic and this can be experienced in different ways. Which option best suits your situation?** |  |  | **<0.001** |  |  | **<0.001** |  |  | **<0.001** | |  |
| *I live about as usual* | 67 (32%) | 63 (18%) |  | 70 (30%) | 78 (19%) |  | 66 (24%) | 52 (13%) |  | |  |
| *I'm more isolated, and it feels mostly difficult* | 60 (29%) | 149 (42%) |  | 66 (29%) | 194 (47%) |  | 99 (35%) | 194 (48%) |  | |  |
| *I'm more isolated, but it does not feel special* | 67 (32%) | 123 (35%) |  | 82 (35%) | 125 (30%) |  | 98 (35%) | 142 (35%) |  | |  |
| *I'm more isolated, and it feels mostly positive* | 14 (7%) | 17 (5%) |  | 13 (6%) | 17 (4%) |  | 16 (6%) | 14 (4%) |  | |  |
| **Life situation affected during the pandemic** |  |  | 0.188 |  |  | 0.308 |  |  | 0.354 | |  |
| *My life is not affected* | 8 (3%) | 14 (4%) |  | 10 (3%) | 7 (2%) |  | 13 (4%) | 12 (3%) |  | |  |
| *I am only slightly affected* | 123 (51%) | 148 (42%) |  | 185 (44%) | 160 (39%) |  | 140 (44%) | 161 (40%) |  | |  |
| *There's a lot in my life that is affected* | 90 (37%) | 159 (45%) |  | 181 (43%) | 196 (47%) |  | 127 (40%) | 167 (42%) |  | |  |
| *Almost everything in my life is affected* | 22 (9%) | 31 (9%) |  | 42 (10%) | 50 (12%) |  | 37 (12%) | 62 (15%) |  | |  |
| *^a^ gestational week.*  ***^b^*** *chi-square derived p-value.* | | | | | | | | | | | |

**Supplementary table 2a.** Prevalence of positive screening for depression, anxiety and wellbeing, by confirmed or possible Covid-19 infection and self-reported impact on life situation.

|  | Covid-19 symptoms^a^ | | | Life situation^a^ | | Social isolation^a^ | | | |
| --- | --- | --- | --- | --- | --- | --- | --- | --- | --- |
| GW 11-20  GW 22-30  GW 32-42 | Yes  n (%) | No  n (%) | Other close friend/family  n (%) | Not affected/  Slightly affected  n (%) | A lot/ Almost everything is affected  n (%) | Live as usual  n (%) | More isolation/  difficult  n (%) | More isolation/  Not special  n (%) | More isolation/  positive  n (%) |
| Prevalence of depression (EPDS) | |  |  |  |  |  |  |  |  |
| gw^b^ 12-22 | 46 (22%) | 51 (22%) | 18 (18%) | 60 (22%) | 56 (20%) | 20 (17%)* | 52 (27%)* | 25 (14%)* | 11 (37%)* |
| gw^b^ 24-34 | 60 (23%) | 67 (21%) | 27 (21%) | 57 (18%) | 100 (24%) | 26 (20%) | 59 (25%) | 32 (17%) | 6 (23%) |
| gw^b^ 36-42 | 30 (16%) | 35 (14%) | 20 (19%) | 36 (14%) | 51 (17%) | 13 (14%)* | 49 (22%)* | 12 (7%)* | 5 (19%)* |
| Prevalence of anxiety (EPDS-3A) | |  |  |  |  |  |  |  |  |
| gw^b^ 12-22 | 14 (7%) | 18 (8%) | 3 (3%) | 15 (6%) | 19 (7%) | 3 (3%)* | 18 (9%)* | 4 (2%)* | 4 (13%)* |
| gw^b^ 24-34 | 16 (6%) | 28 (9%) | 7 (5%) | 15 (5%)* | 37 (9%)* | 11 (9%) | 19 (8%) | 7 (4%) | 1 (4%) |
| gw^b^ 36-42 | 6 (3%) | 10 (4%) | 8 (8%) | 9 (4%) | 17 (6%) | 4 (4%) | 11 (5%) | 3 (2%) | 3 (11%) |
| Prevalence of low well-being (WHO-5) | |  |  |  |  |  |  |  |  |
| gw^b^ 11 | 45 (54%) | 46 (54%) | 19 (49%) | 42 (42%)* | 71 (64%)* | 15 (50%)* | 56 (66%)* | 29 (41%)* | 4 (44%)* |
| gw^b^ 22 | 83 (47%)* | 61 (32%)* | 21 (28%)* | 62 (34%) | 109 (41%) | 33 (38%) | 69 (42%) | 40 (33%) | 2 (14%) |
| gw^b^ 32 | 88 (48%) | 69 (40%) | 31 (39%) | 76 (38%)* | 119 (48%)* | 31 (46%)* | 101 (52%)* | 48 (33%)* | 6 (33%)* |

*^a^ answered at pregnancy weeks 11-20, 22-30 and 32-42;*

*^b^ gestational week;*

** chi-square derived p-value <0.05.*

**Supplementary table 2b. Prevalence of positive screening for depression, anxiety and wellbeing, by confirmed or possible Covid-19 infection and self-reported impact on life situation.**

|  | **Periods**  **1** Jan 2020-Sep 2020  **2** Oct 2020-Feb 2021 | **Covid-19 symptoms^a^** | | | **Life situation^a^** | | **Social isolation^a^** | | | | |
| --- | --- | --- | --- | --- | --- | --- | --- | --- | --- | --- | --- |
|  |  | Yes  n (%) | No  n (%) | Other close friend/  family  n (%) | Not affected/  Slightly affected  n (%) | A lot/ Almost everything is affected  n (%) | Live as usual  n (%) | More isolation/  difficult  n (%) | More isolation/  Not special  n (%) | More isolation/  positive  n (%) |  |
| **Prevalence of depression (EPDS)** |  |  |  |  |  |  |  |  |  |  |  |
| gw^b^ 12-22 | 1 | 14 (29%) | 25 (21%) | 11 (26%) | 30 (25%) | 19 (20%) | 11 (18%)* | 13 (27%)* | 10 (18%)* | 7 (54%)* |  |
|  | 2 | 30 (19%) | 25 (23%) | 7 (13%) | 29 (19%) | 35 (19%) | 9 (16%)* | 37 (26%)* | 14 (12%)* | 4 (25%)* |  |
| gw^b^ 24-34 | 1 | 15 (24%) | 39 (20%) | 18 (23%) | 25 (16%)* | 48 (26%)* | 9 (17%) | 14 (24%) | 12 (17%) | 4 (40%) |  |
|  | 2 | 43 (22%) | 26 (20%) | 9 (19%) | 31 (21%) | 49 (22%) | 16 (23%) | 43 (24%) | 19 (16%) | 2 (13%) |  |
| gw^b^ 36-42 | 1 | 7 (16%) | 18 (14%) | 7 (15%) | 17 (16%) | 15 (13%) | 5 (13%) | 10 (14%) | 5 (8%) | 4 (31%) |  |
|  | 2 | 20 (14%) | 12 (13%) | 13 (27%) | 14 (11%)* | 33 (20%)* | 6 (15%)* | 35 (26%)* | 5 (5%)* | 1 (7%)* |  |
|  |  |  |  |  |  |  |  |  |  |  |  |
| **Prevalence of anxiety (EPDS-A)** |  |  |  |  |  |  |  |  |  |  |  |
| gw^b^ 12-22 | 1 | 7 (14%) | 9 (8%) | 3 (7%) | 8 (7%) | 10 (11%) | 1 (2%)* | 8 (17%)* | 2 (4%)* | 2 (15%)* |  |
|  | 2 | 6 (4%)* | 9 (8%)* | 0 (0%)* | 7 (5%) | 8 (4%) | 2 (4%) | 9 (6%) | 2 (2%) | 2 (13%) |  |
| gw^b^ 24-34 | 1 | 1 (2%) | 19 (10%) | 4 (5%) | 8 (5%) | 16 (9%) | 3 (6%) | 4 (7%) | 3 (4%) | 0 (0%) |  |
|  | 2 | 14 (7%) | 8 (6%) | 3 (6%) | 7 (5%) | 19 (8%) | 7 (10%) | 14 (8%) | 4 (3%) | 1 (7%) |  |
| gw^b^ 36-42 | 1 | 3 (7%) | 6 (5%) | 4 (9%) | 6 (6%) | 7 (6%) | 2 (5%) | 1 (1%) | 3 (5%) | 2 (15%) |  |
|  | 2 | 3 (2%) | 2 (2%) | 4 (8%) | 2 (2%) | 9 (6%) | 1 (3%)* | 9 (7%)* | 0 (0%)* | 1 (7%)* |  |
|  |  |  |  |  |  |  |  |  |  |  |  |
| **Prevalence of low well-being (WHO-5)** |  |  |  |  |  |  |  |  |  |  |  |
| gw^b^ 12-22 | 1 | 18 (62%) | 23 (52%) | 9 (53%) | 20 (43%)* | 30 (70%)* | 8 (47%) | 20 (74%) | 11 (42%) | 2 (67%) |  |
|  | 2 | 27 (50%) | 22 (54%) | 10 (45%) | 22 (42%)* | 40 (60%)* | 7 (54%) | 35 (63%) | 18 (40%) | 2 (33%) |  |
| gw^b^ 24-34 | 1 | 21 (55%)* | 38 (37%)* | 12 (29%)* | 31 (34%) | 41 (45%) | 18 (40%) | 13 (41%) | 13 (33%) | 1 (17%) |  |
|  | 2 | 62 (45%)* | 22 (26%)* | 8 (24%)* | 30 (33%) | 67 (39%) | 15 (36%) | 55 (41%) | 26 (32%) | 1 (13%) |  |
| gw^b^ 36-42 | 1 | 21 (58%)* | 41 (41%)* | 9 (23%)* | 32 (41%) | 39 (39%) | 18 (50%) | 29 (47%) | 13 (26%) | 2 (29%) |  |
|  | 2 | 66 (45%) | 27 (37%) | 22 (55%) | 42 (35%)* | 79 (53%)* | 12 (39%)* | 72 (54%)* | 34 (36%)* | 3 (30%)* |  |

*^a^ answered at pregnancy weeks 11-20, 22-30 and 32-42;*

*^b^ gestational week;*

** chi-square derived p-value <0.05.*
